# A Pragmatic Tele-Nursing Program Improves Satisfaction of Patients with Pulmonary Fibrosis and Their Caregivers—A Pilot Study

**DOI:** 10.3390/medicina61081385

**Published:** 2025-07-30

**Authors:** Mireia Baiges, David Iglesias, Sara Persentili, Marta Jiménez, Pilar Ortega, Jaume Bordas-Martinez

**Affiliations:** 1 Pulmonology Department, Hospital General de Granollers, 08402 Barcelona, Spain; mbaiges@fphag.org (M.B.); mportegac@fphag.org (P.O.); 2 Nursing Department, Hospital General de Granollers, 08402 Barcelona, Spain; diglesias@fphag.org; 3 Universidad de Extremadura—UEX, 06006 Badajoz, Spain; 4 Psychology Department, Hospital General de Granollers, 08402 Barcelona, Spain; espersentili@fphag.org; 5 Patient Experience Department, Hospital General de Granollers, 08402 Barcelona, Spain; mjimenezt@fphag.org; 6 Pulmonology Department, Universidad Internacional de Cataluña—UIC, 08017 Barcelona, Spain

**Keywords:** tele-nursing, pulmonary fibrosis, telemedicine, interstitial lung diseases, patient-reported experience measures, patient-reported outcome measures

## Abstract

*Background and Objectives*: Specialized nurses play an essential role in managing pulmonary fibrosis. While tele-nursing has the potential to optimize disease management, current evidence regarding its impact remains limited. This study aimed to evaluate a tele-nursing intervention that provided unscheduled access to a specialized nurse via phone or email for both patients and caregivers. *Materials and Methods*: This was a prospective, single-center, open-label, and pre–post pilot study. Participants and their caregivers were provided with direct access to a specialized nurse, by phone and email, for unscheduled consultations. Patient-reported experience measures (PREMs) and patient-reported outcome measures (PROMs) were collected at baseline and after three months of tele-nursing access. PREMs were assessed using a 10-point Likert scale questionnaire, and PROMs were evaluated using the King’s Brief Interstitial Lung Disease (K-BILD) and the Living with Pulmonary Fibrosis (L-PF) questionnaires. *Results*: A total of 47 patients with pulmonary fibrosis receiving antifibrotic drugs were enrolled. At three months, 44 patients and 34 caregivers completed the questionnaires. Four patients did not complete the study due to death, lung transplantation, or transition to end-of-life care. No significant changes were observed in PROMs. However, PREMs showed significant improvements, with most scores exceeding 9/10. Patient satisfaction increased by 28% (*p* < 0.001), and caregiver satisfaction by 30% (*p* < 0.001). Caregivers of patients who did not complete the study also reported high satisfaction, comparable to that of other caregivers. *Conclusions*: A pragmatic and affordable tele-nursing program, based on direct phone and email consultations, may enhance patient and caregiver satisfaction in the management of pulmonary fibrosis.

## 1. Introduction

Interstitial lung diseases (ILDs) encompass a broad and heterogeneous group of disorders, many of which may progress to pulmonary fibrosis. The most common is idiopathic pulmonary fibrosis (IPF), a progressive pulmonary condition characterized by progressive decline in lung function, worsening symptoms such as dyspnea and cough, and frequent exacerbations, with a median survival of 3–5 years in the absence of treatment [1,2]. Progressive pulmonary fibrosis (PPF) refers to a similar clinical course in non-IPF fibrosing ILDs, marked by worsening symptoms, functional decline, and radiological progression despite treatment of the underlying ILD [3,4].

Antifibrotic therapies, such as pirfenidone and nintedanib, have demonstrated benefits in both IPF and PPF by slowing the decline in forced vital capacity (FVC), reducing the risk of acute exacerbations, and improving quality of life [5,6,7,8]. Additionally, real-world data have shown improved survival [9]. However, these treatments are often associated with a high incidence of adverse effects, affecting up to two-thirds of patients [5,8,10], which can lead to treatment discontinuation or suboptimal dosing. Beyond antifibrotic therapy, these patients frequently experience symptoms and multiple comorbidities that require a comprehensive and multidisciplinary approach to care [11,12,13].

Specialized nurses play an essential role in managing pulmonary fibrosis by educating patients, handling treatment-related side effects, addressing symptoms, and identifying unmet needs [11]. Their involvement is particularly valuable in complex or evolving clinical scenarios that cannot always be addressed during scheduled medical visits [14]. Given the unpredictable nature of disease-related symptoms and drug-related adverse effects, patients and caregivers often require access to specialized nursing support outside of routine appointments [11].

In this context, telehealth has emerged as a promising tool to extend care beyond the hospital setting, enabling more flexible and responsive models of support [15]. Telemonitoring systems involving smartphone applications, home spirometry, or wearable devices have shown promising outcomes in ILD management [7,8,9]. However, these technologies are often expensive, require complex implementation, and may not be appropriate or feasible for all patient populations, particularly older adults or those with limited familiarity with digital tools. Moreover, it should be taken into account that telemedicine remains largely inaccessible due to limited reimbursement coverage by public healthcare systems [16,17].

Therefore, this study aimed to assess the impact of a tele-nursing intervention on pulmonary fibrosis patients through unscheduled access to a specialized nurse via phone or email by both patients and caregivers.

## 2. Methods

This was a prospective, single-center, open-label, and pre–post pilot study. All consecutive patients with a confirmed diagnosis of ILD (including IPF and non-IPF PPF) who were receiving antifibrotic therapy and attended in-person visits between May and September 2024 were prospectively invited to participate. Therefore, patients not scheduled for in-person visits during this period were not eligible for inclusion.

Inclusion criteria were (a) confirmed ILD diagnosis established by a multidisciplinary committee [1,3] and (b) current treatment with pirfenidone or nintedanib in accordance with international guidelines [1,3]. Exclusion criteria included refusal to participate or inability to complete the questionnaires due to cognitive, linguistic, or physical limitations. No patients were excluded based on disease severity, comorbidities, or prognosis. Patients with a high likelihood of death or lung transplantation within 12 months, in the opinion of the investigator, were not excluded. Caregivers of enrolled patients were also invited to participate.

### 2.1. Nursing Standard Care

Before the intervention, standard care included an initial nurse-led visit for antifibrotic drug initiation, followed by scheduled monthly follow-ups for six months and, subsequently, every three months if no adverse events or management needs were identified. The follow-up was either in person or by phone, with no direct nurse contact for reporting adverse effects or specific health concerns beyond the general hospital helpline, which had no direct access to a specialized nurse.

The initial visit included disease-specific education and a comprehensive assessment covering psychosocial status, family support, symptom burden (dyspnea, fatigue, and cough), nutritional habits, physical activity, bowel function, and hydration. Education was provided on medication administration (e.g., timing and intake of nintedanib), management of potential adverse effects (e.g., diarrhea, nausea, reflux), and dietary strategies. Written materials were provided to reinforce key messages.

Follow-up visits included monitoring of weight, liver function, bowel habits, symptom evolution, and treatment tolerability. The nurse used predefined checklists to guide each visit, ensuring that important points were consistently addressed. Although scripted cues were not used, the structure of the visits was uniform, and patients received tailored advice based on their evolving clinical status.

### 2.2. Study Intervention

The intervention consisted of providing patients and caregivers with direct access to a specialized nurse via telephone and email for unscheduled consultations, available during regular working hours. The rest of the follow-up was conducted as per standard care; scheduled visits were not modified and maintained their usual frequency and format (in-person or remote). The only change introduced was the addition of unscheduled, open access to tele-nursing support between routine visits.

As a second-level hospital, nursing care for ILD patients was carried out by a single specialized nurse. Therefore, all patients in the intervention group were managed by the same nurse, ensuring consistency between the standard care previously provided and the study intervention.

Patient-reported experience measures (PREMs) and patient-reported outcome measures (PROMs) were collected at baseline and after three months of tele-nursing access. PREMs were assessed using a 10-point Likert scale questionnaire, while PROMs were evaluated using the King’s Brief Interstitial Lung Disease (K-BILD) questionnaire and the Living with Pulmonary Fibrosis (L-PF) questionnaire.

### 2.3. Statistical Analysis

Continuous variables are expressed as mean ± standard deviation (SD) for normally distributed data or as median and interquartile range (IQR) for non-normally distributed data. Comparisons between paired observations were performed using the paired Student’s *t*-test for normally distributed variables and the Wilcoxon signed-rank test for non-normally distributed variables. To explore the potential influence of diagnostic heterogeneity on study outcomes, a subgroup analysis was performed, comparing patients with IPF versus PPF (non-IPF). A two-tailed *p*-value < 0.05 was considered statistically significant. All statistical analyses were conducted using STATA software, version 17.0 (StataCorp LLC, College Station, TX, USA).

Due to the exploratory nature of this pilot study, no formal sample size calculation was performed. Instead, all consecutive patients attending the hospital over a four-month period were prospectively invited to participate.

This study was approved by the hospital’s ethics committee (CEIm: 20243004). All participants signed the informed consent form prior to taking part in the study.

## 3. Results

A total of 47 patients with IPF or PPF were enrolled, with no refusals to participate (Table 1). Their mean age was 72.9 years (SD 8.2), and 70% of participants were male. ILD diagnoses included IPF (38%), hypersensitivity pneumonitis (32%), connective tissue disease-associated ILD (11%), unclassifiable ILD (11%), and other types (13%). The mean FVC was 78.7% (SD 0.7), and the mean diffusing capacity for carbon monoxide (DLCO) was 50.5% (SD 15.1) of the predicted value. The average six-minute walk distance (6MWD) was 355.7 m (SD 111.1), and the mean body mass index (BMI) was 27.9 kg/m^2^ (SD 3.6). Severe pulmonary fibrosis, defined as FVC < 50% and/or DLCO < 30%, was present in 23% of patients. Cough was reported by 32% of patients. Dyspnea severity, assessed using the modified Medical Research Council (mMRC) scale, was grade 1 in 43%, grade 2 in 26%, and grade 3 in 13%. All patients were receiving antifibrotic therapy, with 40% reporting treatment-related side effects, mainly gastrointestinal.

At three months, 44 patients and 34 caregivers completed the PREMs and PROMs assessments (Table 2). Four patients did not complete the study due to death (n = 2), lung transplantation (n = 1), or transition to end-of-life care (n = 1).

Among patients, all PREMs showed significant improvements following the implementation of the tele-nursing program, with most scores exceeding 9/10. The most notable benefits were observed in perceived accessibility and availability of the nurse, which improved from a mean of 6.7 (SD 2.6) to 9.4 (SD 1.0) (*p* < 0.001) and from 6.7 (SD 2.6) to 9.6 (SD 0.9) (*p* < 0.001), respectively. Satisfaction with care increased from 7.4 (SD 2.7) to 9.5 (SD 1.0) (*p* < 0.001), while perceived impact of the nurse on health and the importance of remote access also improved significantly. Overall, patient satisfaction increased by 28% (*p* < 0.001). In contrast, PROMs, including the K-BILD and L-PF questionnaires, did not show significant changes after three months.

Caregivers also reported substantial improvements in most PREMs. Ease of access to the nurse improved from 6.8 (SD 2.9) to 9.1 (SD 1.3) (*p* < 0.001), and satisfaction with the care rose from 7.4 (SD 2.7) to 9.6 (SD 0.8) (*p* < 0.001). However, although the perceived importance of remote access to nursing care increased from 9.0 (SD 1.9) to 9.6 (SD 0.9) (*p* = 0.084), and the perceived essential role of the nurse rose from 8.9 (SD 1.9) to 9.5 (SD 1.1) (*p* = 0.096), these changes did not reach statistical significance. Overall, caregiver satisfaction increased by 30% (*p* < 0.001). Caregivers of patients who discontinued the study also reported high satisfaction with tele-nursing, comparable to that of other caregivers.

A subgroup analysis was conducted to explore potential differences in outcomes between diagnostic categories (IPF vs. non-IPF). The analysis included outcomes of both patients and their respective caregivers according to ILD diagnostics. No statistically significant differences were observed between the IPF and non-IPF groups in any of the assessed outcomes.

## 4. Discussion

This study highlights the potential role of tele-nursing in improving patient and caregiver satisfaction and experience in pulmonary fibrosis management. Tele-nursing, implemented through a simple and cost-effective model based on phone and email consultations, offers a feasible and scalable approach to enhance care, even in advanced stages of the disease. This is particularly relevant given the complexity of managing pulmonary fibrosis, a condition often associated with unmet care needs, including the need for clinical assessment outside of scheduled appointments [13].

Our significant improvement in PREMs, despite the lack of meaningful changes in PROMs, is consistent with findings from previous telehealth interventions in patients with ILD. Moore et al. [18] conducted a multicenter randomized controlled trial in IPF patients, evaluating a 24-week home monitoring program that incorporated digital tools such as home spirometry, symptom and side effect reporting, patient-reported outcomes, educational content, a medication coach, and eConsultations. This intervention facilitated early detection of clinical deterioration and, similar to our findings, led to improved patient satisfaction without significant changes in K-BILD scores. Likewise, a pilot study in patients with systemic sclerosis-associated ILD using a smartphone-integrated home spirometer over six weeks demonstrated high adherence and satisfaction; however, PROMs results were heterogeneous, with significant improvement in K-BILD and EQ-5D-5L index value but no differences in EQ-5D visual analogue scale score, HAD-anxiety, or HAD-depression scores [19]. In the FACT study [20], a 12-week home-based spirometry program in ILD patients showed high feasibility and adherence, yet PROMs (K-BILD) remained unchanged. Nevertheless, participants reported increased disease awareness and a sense of empowerment, underscoring the value of incorporating patient experience measures when assessing telehealth interventions in ILD.

The limited improvement observed in PROMs in our study is likely multifactorial. As with other non-physical interventions, tele-nursing tends to have minimal impact on clinical outcomes and health-related quality of life (HRQoL) [21], while improvements in PREMs are more consistently reported across chronic disease populations. One contributing factor may be the short duration of follow-up, which has previously been identified as a potential reason for the failure to detect significant changes in HRQoL, despite measurable benefits in other clinical parameters such as lung function [22]. Moreover, the 2025 research statement from the American Thoracic Society highlights the methodological challenges in interpreting PROMs within home-based monitoring studies and recommends the development of more robust tools to better assess patient-centered outcomes in ILD [23]. Supporting this, a recent systematic review of PROMs for cough in ILD concluded that currently available instruments lack sufficient validation for this population [24].

To explore potential differences in response to the intervention across ILD subtypes, a subgroup analysis was performed comparing patients with IPF and those with PPF (non-IPF). The absence of variation in patient and caregiver-reported outcomes suggests that the benefits of nurse-led telehealth support may be broadly applicable across the spectrum of progressive fibrotic ILDs. This likely reflects the shared clinical features among these conditions, including symptom burden, functional decline, and psychosocial stressors. The findings are consistent with the broader understanding that patients with fibrotic ILDs, regardless of etiology, face similar challenges such as breathlessness, fatigue, emotional distress, and unmet supportive care needs [13,25,26].

Beyond clinical outcomes, our study also addresses structural barriers to care. Despite increasing evidence supporting the effectiveness of telehealth interventions in the management of chronic lung diseases [27,28], access to such services remains limited in many European regions, primarily due to the absence of reimbursement mechanisms within public healthcare systems [29]. In our study, this barrier was addressed through the implementation of a pragmatic tele-nursing model based on telephone and email consultations, which enabled patients to access a specialized nurse directly and on an unscheduled basis. This approach significantly enhanced their overall experience and satisfaction with care.

This is particularly relevant in underserved or rural settings. Boente et al. [30] evaluated a web-based home monitoring program including home spirometry for rural patients with ILD and identified reductions in access barriers such as travel time, distance, and associated costs as the most notable perceived benefits. However, the study also highlighted persistent challenges to telehealth implementation, including limited funding, lack of reimbursement, and inadequate internet connectivity. In this context, the positive impact observed in our study on patient satisfaction and perceived experience reinforces the value of integrating tele-nursing into routine care, particularly in settings with restricted access to in-person consultations. Furthermore, given the disparities in healthcare resource availability, even within the same country [29], the implementation of pragmatic and cost-effective telemedicine models like ours offers a promising and scalable solution.

In addition to overall improvement, PREMs in our study showed reduced variability (SD), suggesting that patients with previously unmet needs experienced the greatest benefit. This highlights the importance of targeted care [14], as those with the highest support needs gained the most from direct tele-nursing access. Notably, the most significant improvement was observed in patients’ ability to easily contact the nurse when needed.

For caregivers, the most meaningful change was the sense of security derived from having direct access to a specialized nurse, without the uncertainty of navigating a general hospital helpline. Although changes in caregivers’ ratings regarding the importance of remote access and the essential role of the nurse did not reach statistical significance, this likely reflects the already high baseline scores. These aspects were clearly valued from the outset, and the availability of tele-nursing may have reinforced these strong initial perceptions. The importance of direct communication became especially evident during the COVID-19 pandemic, when the use of telephone consultations increased significantly [31]. According to our caregivers’ responses, this remains relevant today. Moreover, phone-based telemedicine may help reduce barriers to care and promote equity, particularly among older adults and digitally excluded populations [16].

Finally, attrition due to death, lung transplantation, or transition to end-of-life care affected four participants in our study, underscoring the clinical fragility of this population and the importance of supportive interventions that remain viable in advanced disease stages. Notably, caregivers of these patients reported high levels of satisfaction with the tele-nursing program, reinforcing its value in providing reassurance and continuity of care during critical transitions. This observation reflects a broader understanding of the emotional and informational demands placed on caregivers in advanced ILD [13,25,26]. Including tele-nursing as part of regular care for patients in advanced stages may help improve early guidance, symptom management, and emotional support [21,22,23]. Its simplicity and flexibility make it a practical option for reaching patients and families who face barriers to in-person care.

### 4.1. Strengths and Limitations

This study presents limitations that must be acknowledged. First, the lack of a control group and the open-label design limit the ability to infer causality between the intervention and the observed outcomes. This limitation was anticipated and reflects the exploratory nature of a pilot study, which was designed as a proof of concept to assess feasibility and acceptability, in order to avoid unnecessary patient burden and inefficient use of resources. A subsequent randomized controlled study will be necessary to validate these findings and confirm their generalizability. Second, the short follow-up period (three months) limits the assessment of long-term effects on clinical outcomes, patient behavior, and sustained satisfaction. Third, the single-center design and relatively small sample size may limit the generalizability of the findings to other healthcare settings or populations. Finally, the study relied predominantly on subjective measures (PREMs and PROMs), which may introduce response bias.

Despite these limitations, the study has notable strengths. The study reflects real-world clinical practice and evaluates a low-cost, easily implementable tele-nursing model that does not depend on advanced technology nor require significant investment. The inclusion of both patients and caregivers allows for a more comprehensive assessment of the intervention’s impact. This is particularly relevant given the limited existing evidence on the impact of tele-nursing in patients with pulmonary fibrosis. Finally, the inclusion of different ILD diagnoses enhances the generalizability of the intervention and reflects the heterogeneity encountered in real-world clinical practice.

### 4.2. Practical Implications

This tele-nursing model is defined by its simplicity, low cost, and ease of implementation. It requires no investment in digital platforms, remote monitoring devices, or specialized software. Instead, it relies solely on the availability of a trained nurse, a telephone line, and an institutional email account. As such, it can be readily adopted by any ILD unit, regardless of size or technological infrastructure. Any center with a dedicated ILD nurse could implement this model immediately, without the need for additional resources or structural changes. Although initially implemented in a hospital-based ILD unit, its minimal resource requirements and operational flexibility make it a highly scalable solution, easily adaptable to primary care, rural settings, or healthcare systems with limited digital infrastructure. Its integration into routine clinical care makes it a scalable and accessible strategy to enhance support for both patients and caregivers in real-world settings.

### 4.3. Study Implications and Future Research

This study supports the integration of a pragmatic and low-complexity tele-nursing model into routine care for patients with pulmonary fibrosis. The intervention, based on direct phone and email access to a specialized nurse, proved feasible, cost-effective, and well accepted by both patients and caregivers. These findings are particularly relevant in healthcare settings with limited access to in-person care or digital infrastructure. By showing that even minimal interventions can result in high satisfaction, the study underscores the importance of enhancing communication and support in chronic disease management. Future research should validate these findings in larger, multicenter cohorts with extended follow-up periods. It should also evaluate the impact of tele-nursing on clinical outcomes, caregiver burden, healthcare utilization, and cost-effectiveness, incorporating objective endpoints and considerations of equity in access.

## 5. Conclusions

Our study suggests that a pragmatic and affordable tele-nursing program, based on direct phone and email consultations, may enhance patient and caregiver satisfaction in the management of pulmonary fibrosis. Future studies with larger, multicenter cohorts and longer follow-up periods are needed to explore the long-term effects of tele-nursing on both clinical outcomes and quality of life.

## Figures and Tables

**Table 1:** Demographic and clinical characteristics.

Characteristics		N = 47
Male, n (%)		33 (70%)
Age, mean (SD)		72.9 (8.2)
Diagnosis, n (%)	IPF	18 (38%)
	Hypersensitivity pneumonitis	15 (32%)
	Connective tissue disease-associated	5 (11%)
	Unclassifiable	3 (6%)
	Other types	6 (13%)
Severe pulmonary fibrosis, n (%)	FVC < 50% and/or DLCO < 30%	11 (23%)
Cough, n (%)		15 (32%)
Dyspnoea mMRC, n (%)	Grade 0	9 (19%)
	Grade 1	20 (43%)
	Grade 2	12 (26%)
	Grade 3	6 (13%)
Antifibrotic side effects, n (%)		19 (40%)
BMI, mean (SD)		27.9 (3.6)
FVC (L), mean (SD)		3.2 (0.7)
FVC (%), mean (SD)		78.7 (18.9)
DLCO (%), mean (SD)		50.5 (15.1)
6MWT (m), mean (SD)		355.7 (111.1)

SD: standard deviation; IPF: idiopathic pulmonary fibrosis; mMRC: modified Medical Research Council; BMI: body mass index; FVC: forced vital capacity; DLCO: diffusing capacity of the lung for carbon monoxide; 6MWT: 6-Minute Walk Test.

**Table 2:** PREMs and PROMs of tele-nursing.

PATIENT					N
	PREMs	Basal	After	*p*-Value	
	(I feel)	Mean (SD)	Mean (SD)		
1	Having a nurse is essential for my care	9.1 (1.3)	9.8 (0.7)	0.004	43
2	Access to the nurse is easy	6.7 (2.6)	9.4 (1.0)	0.000	43
3	Access to the nurse has been available when I needed it	6.7 (2.6)	9.6 (0.9)	0.000	43
4	All necessary information on symptom management has been provided	7.2 (2.3)	9.4 (1.0)	0.000	43
5	Security in all forms of contact with the nurse	7 (2.4)	9.3 (1.4)	0.000	43
6	The healthcare service is well organized	6.9 (2.7)	9.2 (1.2)	0.000	43
7	Concern for my well-being and issues	7.7 (2.6)	9.6 (0.8)	0.000	43
8	Resolution of my lung disease	7 (2.6)	9.3 (1.2)	0.000	43
9	Good management of symptoms	7.1 (2.5)	8.8 (1.3)	0.000	43
10	Comforted by the attention received	7.2 (2.7)	9.4 (1.1)	0.000	43
11	Self-control over the disease and its treatment	6.9 (2.8)	8.7 (1.6)	0.000	43
12	Rate satisfaction with the care received	7.4 (2.7)	9.5 (1.0)	0.000	43
13	Rate the nurse’s impact on health	8 (1.8)	9 (1.6)	0.007	43
14	Rate the importance of remote access to nurse	8.4 (2.0)	9.7 (0.8)	0.000	43
	PROMs				
	K-BILD	53.9 (9.8)	53.4 (15.0)	0.797	37
	L-PF Impact	48.7 (24.6)	45.3 (22.9)	0.219	41
	L-PF Symptoms	30.9 (21.1)	30.6 (22.1)	0.912	41
**CAREGIVER**					
1	Having a nurse is essential for his/her care	8.9 (1.9)	9.5 (1.1)	0.096	34
2	Access to the nurse is easy	6.8 (2.9)	9.1 (1.3)	0.000	34
3	Access to the nurse has been available when he/she needed it	6.9 (2.8)	9.3 (1.3)	0.000	34
4	All necessary information on symptom management has been provided	7.4 (2.8)	9.3 (1.2)	0.001	34
5	Security in all forms of contact with the nurse	6.7 (3.0)	9.2 (1.4)	0.000	34
6	The healthcare service is well organized	7 (2.9)	9.4 (1.0)	0.000	34
7	Concern for his/her well-being and issues	7.5 (2.9)	9.5 (0.9)	0.000	34
8	Resolution of his/her lung disease	7.1 (2.7)	9.3 (1.2)	0.000	34
9	Good management of his/her symptoms	7.1 (2.6)	8.7 (1.3)	0.000	34
10	Comforted by the attention received	7.2 (2.8)	9.5 (1.0)	0.000	34
11	Self-control over the disease and its treatment	6.5 (2.8)	8.6 (1.5)	0.000	34
12	Rate satisfaction with the care received	7.4 (2.7)	9.6 (0.8)	0.000	34
13	Rate the nurse’s impact on health	8 (2.2)	9.3 (1.3)	0.003	34
14	Rate the importance of remote access to nurse	9.0 (1.9)	9.6 (0.9)	0.084	34

PROMs: patient-reported outcome measures; PREMs: patient-reported experience measures; SD: standard deviation; K-BILD: King’s Brief Interstitial Lung Disease questionnaire; L-PF: Living with Pulmonary Fibrosis questionnaire.

## Data Availability

The data that support the findings of this study are available from the corresponding author upon reasonable request.

## Abbreviations

IPF	idiopathic pulmonary fibrosis
ILD	interstitial lung disease
PPF	progressive pulmonary fibrosis
FVC	forced vital capacity
DLCO	diffusing capacity of the lung for carbon monoxide
BMI	body mass index
6MWD	six-minute walk distance
mMRC	modified Medical Research Council
PREMs	patient-reported experience measures
PROMs	patient-reported outcome measures
K-BILD	King’s Brief Interstitial Lung Disease questionnaire
L-PF	Living with Pulmonary Fibrosis questionnaire
HRQOL	health-related quality of life
IQR	interquartile range
SD	standard deviation
CEIm	research ethics committee
